# Real-world *in vitro* activity of newer antibiotics against Enterobacterales and *Pseudomonas aeruginosa*, including carbapenem-non-susceptible and multidrug-resistant isolates: a multicenter analysis

**DOI:** 10.1128/spectrum.03129-23

**Published:** 2023-11-08

**Authors:** Todd Riccobene, ChinEn Ai, Kalvin C. Yu, Sara Gregory, Brooke Kim, Dmitri Debabov, Vikas Gupta

**Affiliations:** 1 Medical Affiars, AbbVie, Florham Park, New Jersey, USA; 2 Becton, Dickinson and Company (BD), Franklin Lakes, New Jersey, USA; 3 Clinical Microbiology, AbbVie, Irvine, California, USA; JMI Laboratories, North Liberty, Iowa, USA

**Keywords:** ceftazidime–avibactam, carbapenem resistance, Enterobacterales, *Pseudomonas aeruginosa*, antibiotic resistance, multidrug resistance, Gram-negative pathogens

## Abstract

New antibiotics have been developed to combat antibiotic-resistant Gram-negative pathogens, which are difficult to treat and associated with poor clinical outcomes. We conducted a multicenter evaluation of real-world testing practices and susceptibility to newer antibiotics. Our study included 71 facilities in the BD Insights Research Database (2018–2022) and involved adult patients with a positive culture for Enterobacterales or *Pseudomonas aeruginosa* and facility-reported antibiotic susceptibility data for cefiderocol (FDC), ceftazidime–avibactam (CZA), ceftolozane–tazobactam (C/T), eravacycline (ERV), imipenem–relabactam (I-R), or meropenem–vaborbactam (MVB). A total of 27,531 susceptibility results were available, including 22,111 (80.3%) Enterobacterales and 5,420 (19.7%) *P*. *aeruginosa. Escherichia coli* (11,882 [43.2%]) was the most frequently tested potential pathogen, and CZA (13,567 [49.3%]) and C/T (13,299 [48.3%]) had the greatest numbers of susceptibility results. For the other four antibiotics, susceptibility data were available for fewer than 2% of isolates. Susceptibility comparisons should be considered with caution. Among isolates tested, CZA had the highest susceptibility rates for total Enterobacterales (98.7%) and multidrug-resistant (92.9%) and carbapenem-non-susceptible (85.0%) isolates. For *P. aeruginosa*, FDC had the highest susceptibility rates for total (95.6%) and carbapenem-non-susceptible (93.3%) isolates. The susceptibility of carbapenem-non-susceptible Enterobacterales to CZA and C/T decreased modestly from 2020 to 2022, but carbapenem-non-susceptible *P. aeruginosa* susceptibility rates increased. We conclude that Gram-negative pathogens have high real-world susceptibility rates to newer antibiotics but may show important differences across resistance profiles. Newer antibiotics are a valuable option for the management of resistant Enterobacterales and *P. aeruginosa*. Additional effort may be required to integrate these agents into routine clinical care.

**IMPORTANCE:**

Newer antibiotics against Gram-negative pathogens provide important treatment options, especially for antibiotic-resistant bacteria, but little is known about their use during routine clinical care. To use these agents appropriately, clinicians need to have access to timely susceptibility data. We evaluated 27,531 facility-reported susceptibility results from the BD Insights Research Database to gain a better understanding of real-world testing practices and susceptibility rates for six newer antibiotics. *Escherichia coli* was the most frequently tested potential pathogen, and ceftazidime–avibactam and ceftolozane–tazobactam had the greatest numbers of susceptibility results. For cefiderocol, eravacycline, imipenem–relabactam, and meropenem–vaborbactam, susceptibility data were available for fewer than 2% of isolates. Susceptibility comparisons should be considered with caution. Ceftazidime–avibactam had the highest susceptibility rates for Enterobacterales while cefiderocol had the highest susceptibility rates for *Pseudomonas aeruginosa*. New antibiotics have the potential to improve the management of Gram-negative infections, but their use may be hampered by the absence of susceptibility data.

## INTRODUCTION

Infections caused by antimicrobial-resistant (AMR) Gram-negative bacteria are increasingly problematic in healthcare settings and are associated with worse clinical outcomes in critically ill patients (1 – 4). Several categories of AMR Gram-negative pathogens are considered significant threats by the US Centers for Disease Control and Prevention and the World Health Organization, including carbapenem-resistant Enterobacterales, extended-spectrum beta-lactamase-producing Enterobacterales, carbapenem-resistant *Pseudomonas aeruginosa*, and multidrug-resistant *P. aeruginosa* (5, 6). AMR *Escherichia coli*, the leading pathogen in terms of deaths, was estimated to be associated with approximately 800,000 deaths worldwide in 2019, and *Klebsiella pneumoniae*, the third leading AMR pathogen, was associated with over 600,000 deaths (1). These stark numbers have led to increased efforts to develop new drugs with activity against AMR Gram-negative pathogens and optimize the use of existing therapeutic options in patients with these infections.

In the past 10 years, several newer antibiotics have been approved by the US Food and Drug Administration (FDA) to address this need. These antibiotics vary in their specific activity profiles, particularly with respect to carbapenem-resistant phenotypes of Enterobacterales, but all have shown high levels of activity against key Gram-negative pathogens (7, 8). Surveillance studies conducted at central laboratories have played a vital role in providing susceptibility data on newer antibiotics across large numbers of pathogens and clinical indications (9 – 11). However, they are not designed to address issues involved in real-world use, such as which isolates undergo susceptibility testing for these drugs during routine clinical care and what susceptibility rates are observed under conditions where the drugs are being considered for use.

We used data from electronic health records to conduct a multicenter evaluation of susceptibilities to newer antibiotics in hospitalized US patients. The goal of this study was to evaluate real-world susceptibility testing practices and profiles for newer antibiotics with activity against AMR Gram-negative pathogens in order to gain greater insights into the use of these drugs in routine clinical care.

## RESULTS

We evaluated antibiotic susceptibility results for non-duplicate positive cultures of Enterobacterales or *P. aeruginosa* reported from patients at 71 acute care facilities in the US between 2018 and 2022. Antibiotic susceptitablbility analyses included six antibiotics with activity against AMR Gram-negative pathogens: cefiderocol (FDC), ceftazidime–avibactam (CZA), ceftolozane–tazobactam (C/T), eravacycline (ERV), imipenem–relabactam (I-R), and meropenem–vaborbactam (MVB). Culture sources included urine, skin/wound, blood, respiratory, intra-abdominal, and other sources.

### Susceptibility testing by bacterial species and antibiotic

Susceptibility data for one or more newer antibiotics were available for 27,531 isolates, including 22,111 (80.3%) Enterobacterales and 5,420 (19.7%) *P*. *aeruginosa* isolates. Overall, the species with the greatest number of susceptibility results was *E. coli* (11,882 [43.2%]), followed by *P. aeruginosa* (5,420 [19.7%]) and *K. pneumoniae* (4,042 [14.7%]) (Table 1).

**TABLE 1:** Bacterial isolates tested for susceptibility to newer antibiotics by species^*a*^

Bacteria	*n* (% of isolates tested for the specified antibiotic)						Total isolates *n* (% of all isolates)
	CZA	C/T	FDC	MVB	ERV	I-R	
Total isolates with susceptibility results	13,567	13,299	332	226	105	2	27,531
*Escherichia coli*	6,176 (45.5%)	5,561 (41.8%)	4 (1.2%)	81 (35.8%)	60 (57.1%)	−^*b*^	11,882 (43.2%)
*Pseudomonas aeruginosa*	1,900 (14.0%)	3,172 (23.9%)	317 (95.5%)	15 (6.6%)	14 (13.3%)	2 (100%)	5,420 (19.7%)
*Klebsiella pneumoniae*	2,229 (16.4%)	1,734 (13.0%)	4 (1.2%)	56 (24.8%)	19 (18.1%)	−	4,042 (14.7%)
*Proteus mirabilis*	1,266 (9.3%)	965 (7.3%)	−	19 (8.4%)	−	−	2,250 (8.2%)
*Enterobacter cloacae*	652 (4.8%)	632 (4.8%)	2 (0.6%)	28 (12.4%)	10 (9.5%)	−	1,324 (4.8%)
*Serratia marcescens*	345 (2.5%)	266 (2.0%)	3 (0.9%)	7 (3.1%)	−	−	621 (2.3%)
*Klebsiella oxytoca*	287 (2.1%)	260 (2.0%)	−	3 (1.3%)	1 (1.0%)	−	551 (2.0%)
*Klebsiella aerogenes*	237 (1.7%)	249 (1.9%)	2 (0.6%)	4 (1.8%)	1 (1.0%)	−	493 (1.8%)
*Morganella morganii*	211 (1.6%)	172 (1.3%)	−	7 (3.1%)	−	−	390 (1.4%)
*Citrobacter freundii*	173 (1.3%)	178 (1.3%)	−	2 (0.9%)	−	−	353 (1.3%)
*Providencia stuartii*	91 (0.7%)	110 (0.8%)	−	4 (1.8%)	−	−	205 (0.7%)

^*a*^ C/T, ceftolozane-tazobactam; CZA, ceftazidime-avibactam; ERV, eravacycline; FDC, cefiderocol; I-R, imipenem-relabactam; MVB, meropenem-vaborbactam.

^*b*^ "-" indicates that there were no isolates tested for that antibiotic.

Of the six antibiotics evaluated in this study, susceptibility to CZA and C/T was evaluated most frequently (13,567 isolates [49.3%] and 13,299 isolates [48.3%], respectively) (Table 1). For the other four antibiotics, susceptibility data were available for fewer than 2% of isolates (FDC 332 [1.2%]; MVB 226 [0.8%], ERV 105 [0.4%], and I-R 2 [0.007%]). CZA and C/T generally showed similar patterns with respect to species with available susceptibility data, although the proportion of *P. aeruginosa* isolates tested was lower for CZA compared with C/T (14.0% vs 23.9%) (Table 1). FDC and I-R susceptibilities were tested almost exclusively in *P. aeruginosa*, while MVB and ERV susceptibilities were evaluated mostly in Enterobacterales.

### Antibiotic susceptibility rates

*In vitro* antibiotic susceptibility rates to newer antibiotics were evaluated for total isolates with available data and for resistance sub-classes (non-multidrug-resistant [non-MDR], MDR, carbapenem-non-susceptible [Carb-NS], and Carb-NS + MDR) (Table 2). For Enterobacterales, the proportions of MDR and Carb-NS isolates were approximately 15% and 7%, respectively. Most Carb-NS Enterobacterales isolates were also MDR (763/860 [88.7%] for isolates with CZA susceptibility data and 516/653 [79.0%] for isolates with C/T susceptibility data). Because antimicrobial susceptibility testing guidelines recommend that susceptibility data should only be evaluated when at least 30 isolates are available (12), I-R was not included.

**TABLE 2:** *In vitro* susceptibilities to newer antibiotics in hospitalized adult patients with positive cultures for Enterobacterales or *P. aeruginosa* ^*a*,*b*^

Organism group	Resistance type	% of isolates (*n*/*N*)				
		CZA % (*n*/*N*)	C/T % (*n*/*N*)	FDC % (*n*/*N*)	MVB % (*n*/*N*)	ERV % (*n*/*N*)
Enterobacterales	Non-MDR	99.8% (9,798/9,815)	97.4% (8,377/8,598)	<30 isolates	100% (132/132)	98.5% (67/68)
	MDR	92.9% (1,720/1,852)	65.0% (994/1,529)	<30 isolates	86.1% (68/79)	<30 isolates
	Carb-NS	85.0% (731/860)	34.6% (226/653)	<30 isolates	83.6% (56/67)	<30 isolates
	Carb NS + MDR	84.0% (641/763)	20.3% (105/516)	<30 isolates	81.7% (49/60)	<30 isolates
	Total	98.7% (11,518/11,667)	92.5% (9,371/10,127)	<30 isolates	94.8% (200/211)	94.5% (86/91)
*P. aeruginosa*	Non-MDR	98.0% (1,134/1,157)	97.9% (1,365/1,394)	99.0% (99/100)	<30 isolates	<30 isolates
	MDR	70.1% (521/743)	82.1% (1,459/1,778)	94.0% (204/217)	<30 isolates	<30 isolates
	Carb-NS	74.8% (631/844)	84.3% (1,624/1,927)	93.3% (196/210)	<30 isolates	<30 isolates
	Carb-NS + MDR	67.8% (434/640)	80.2% (1,211/1,510)	92.9% (169/182)	<30 isolates	<30 isolates
	Total	87.1% (1,655/1,900)	89.0% (2,824/3,172)	95.6% (303/317)	<30 isolates	<30 isolates

^*a*^ Data are shown for antibiotics with data for ≥30 isolates of Enterobacterales or *P. aeruginosa*. FDC was tested in 15 Enterobacterales isolates, MVB was tested in 15 *P*. *aeruginosa* isolates, and ERV was tested in 15 MDR and 23 Carb-NS isolates.

^*b*^ Carb-NS, carbapenem nonsusceptible; C/T, ceftolozane-tazobactam; CZA, ceftazidime-avibactam; ERV, eravacycline; FDC, cefiderocol; MDR, multidrug resistant; MVB, meropenem-vaborbactam.

Comparisons of susceptibility rates must be viewed with caution, as not all antibiotics were tested in all isolates, and cascade testing (restriction of isolate testing to those shown to be resistant to the prior antibiotic tested) may have influenced results. Among isolates tested in this study, CZA had the highest susceptibility rates for total Enterobacterales (11,518/11,667 [98.7%]), MDR (1,720/1,852 [92.9%]), Carb-NS (731/860 [85.0%]), and Carb-NS +MDR isolates (641/763 [84.0%]) (Table 2). C/T also showed high susceptibility rates for total Enterobacterales isolates (9,371/10,127 [92.5%]), but lower rates for MDR (994/1,529 [65.0%]), Carb-NS (226/653 [34.6%]), and Carb NS +MDR isolates (105/516 [20.3%]). MVB and ERV had high and similar susceptibility rates for total Enterobacterales isolates (200/211 [94.8%] and 86/91 [94.5%], respectively), and MVB had susceptibility rates of >80% for MDR, Carb-NS, and Carb-NS + MDR isolates. There were too few isolates to evaluate these resistance categories for ERV and FDC.

For *P. aeruginosa*, the proportions of MDR and Carb-NS isolates were higher for isolates with C/T susceptibility data compared to those with CZA susceptibility data (approximately 60% versus 40%). For both drugs, about three-quarters of Carb-NS isolates were also MDR. The highest susceptibility rates against *P. aeruginosa* overall and for resistance subgroups were observed for FDC, followed by C/T and CZA (Table 2).

Susceptibility analyses by culture source revealed generally similar susceptibility rates for a given drug across different culture sources (Table 3). A notable exception was the susceptibility of Enterobacterales respiratory isolates to C/T, which was lower than overall C/T susceptibility for total isolates (79.7% versus 92.5%) and Carb-NS isolates (25.4% versus 34.6%). Carb-NS *P. aeruginosa* blood isolates showed a lower susceptibility to CZA (65.6%) compared with all Carb-NS *P. aeruginosa* isolates (74.8%), but the number of blood isolates was small (*n* = 32).

**TABLE 3:** *In vitro* susceptibilities to newer antibiotics in hospitalized adult patients with positive cultures for Enterobacterales or *P. aeruginosa* by culture source^*a*,*b*^

Organism group	Culture source	% of isolates (*n*/*N*)				
		CZA % (*n*/*N*)	C/T % (*n*/*N*)	FDC % (*n*/*N*)	MVB % (*n*/*N*)	ERV % (*n*/*N*)
All isolates						
Enterobacterales	All sources	98.7% (11,518/11,667)	92.5% (9,371/10,127)	<30 isolates	94.8% (200/211)	94.5% (86/91)
	Urine	99.4% (7,083/7,129)	94.2% (5,869/6,228)	<30 isolates	97.1% (101/104)	98.1% (52/53)
	Skin/wound	98.1% (1,588/1618)	87.9% (1,120/1274)	<30 isolates	94.1% (32/34)	<30 isolates
	Blood	99.4% (1,441/1,449)	95.7% (1,293/1,351)	<30 isolates	96.2% (25/26)	<30 isolates
	Respiratory	94.6% (831/878)	79.7% (585/734)	<30 isolates	<30 isolates	<30 isolates
	IA	96.5% (247/256)	94.1% (253/269)	<30 isolates	<30 isolates	<30 isolates
	Other	97.3% (328/337)	92.6% (251/271)	<30 isolates	<30 isolates	<30 isolates
*P. aeruginosa*	All sources	87.1% (1,655/1,900)	89.0% (2,824/3,172)	95.6% (303/317)	<30 isolates	<30 isolates
	Urine	91.2% (498/546)	93.1% (786/844)	100% (53/53)	<30 isolates	<30 isolates
	Skin/wound	88.6% (504/569)	91.4% (761/833)	97.2% (69/71)	<30 isolates	<30 isolates
	Blood	96.2% (75/87)	91.9% (113/123)	<30 isolates	<30 isolates	<30 isolates
	Respiratory	82.3% (519/631)	84.4% (1,048/1,242)	93.1% (163/175)	<30 isolates	<30 isolates
	IA	<30 isolates	<30 isolates	<30 isolates	<30 isolates	<30 isolates
	Other	87.3% (48/55)	89.2% (99/111)	<30 isolates	<30 isolates	<30 isolates
Carb-NS isolates						
Enterobacterales	All sources	85.0% (731/860)	34.6% (226/653)	<30 isolates	83.6% (56/67)	<30 isolates
	Urine	90.2% (304/337)	34.2% (104/304)	<30 isolates		<30 isolates
	Skin/wound	85.6% (167/195)	39.9% (57/143)	<30 isolates	<30 isolates	<30 isolates
	Blood	89.9% (71/79)	40.7% (24/59)	<30 isolates	<30 isolates	<30 isolates
	Respiratory	77.7% (151/193)	25.4% (30/118)	<30 isolates	<30 isolates	<30 isolates
	IA	62.5% (15/24)	<30 isolates	<30 isolates	<30 isolates	<30 isolates
	Other	71.9% (23/32)	<30 isolates	<30 isolates	<30 isolates	<30 isolates
*P. aeruginosa*	All sources	74.8% (631/844)	84.3% (1,624/1,927)	93.3% (196/210)	<30 isolates	<30 isolates
	Urine	77.2% (142/184)	88.7% (407/159)	100% (35/35)	<30 isolates	<30 isolates
	Skin/wound	75.5% (160/212)	86.5% (397/459)	94.4% (34/36)	<30 isolates	<30 isolates
	Blood	65.6% (21/32)	86.4% (51/59)	<30 isolates	<30 isolates	<30 isolates
	Respiratory	74.2% (291/392)	80.5% (712/884)	90.9% (120/132)	<30 isolates	<30 isolates
	IA	<30 isolates	<30 isolates	<30 isolates	<30 isolates	<30 isolates
	Other	<30 isolates	87.0% (47/54)	<30 isolates	<30 isolates	<30 isolates

^*a*^ Data are shown for antibiotics with data for ≥30 isolates of Enterobacterales or *P. aeruginosa* for each culture source. The drugs evaluated here did not have an FDA-approved indication for skin/soft tissue infections at the time of this study.

^*b*^ Carb-NS, carbapenem nonsusceptible; C/T, ceftolozane-tazobactam; CZA, ceftazidime-avibactam; ERV, eravacycline; FDC, cefiderocol; IA, intra-abdominal; MVB, meropenem-vaborbactam.

### Antibiotic susceptibility trends

**Fig 1:**
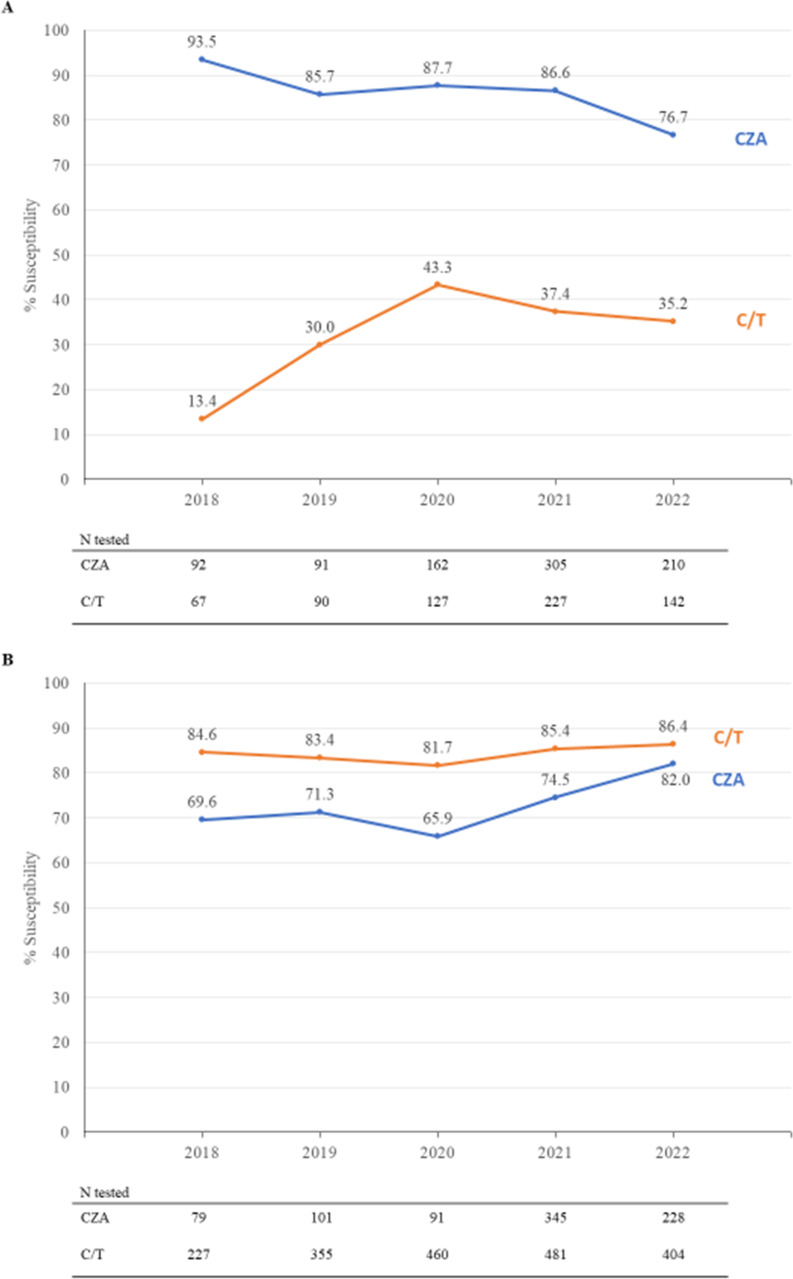
Trends from 2018 to 2022 in ceftazidime–avibactam (CZA) and ceftolozane–tazobactam (C/T) susceptibilities for hospitalized adult patients with (**A**) carbapenem-non-susceptible Enterobacterales and (**B**) carbapenem-non-susceptible *P. aeruginosa*.

An evaluation of trends in antibiotic susceptibility from 2018 to 2022 found that Carb-NS Enterobacterales susceptibility rates were lower for CZA in 2022 (76.7%) than in 2018 (93.5%) but were consistent from 2019 to 2021 (85.7%–87.7%). C/T susceptibility rates were consistently lower than CZA rates during this time period (Fig. 1). Both drugs showed an approximately 10% decrease in Carb-NS Enterobacterales susceptibility rates between 2020 and 2022. For Carb-NS *P. aeruginosa*, CZA susceptibility rates increased over time and reached their highest levels in 2022 (82.0% compared to 86.4% for C/T). The susceptibility of *P. aeruginosa* to C/T stayed fairly constant over this time period (84.6% in 2018 and 86.4% in 2022) (Fig. 1). For both Enterobacterales and *P. aeruginosa*, 2021 was the year in which the most isolates were tested.

## DISCUSSION

Our multicenter study of hospitalized patients with positive cultures for Enterobacterales or *P. aeruginosa* found that about 80% of isolates tested for susceptibility to newer antibiotics were Enterobacterales, most commonly *E. coli*, and about 20% were *P. aeruginosa*. Of the six drugs evaluated, the most frequently tested drugs were CZA and C/T. These two drugs also had the earliest approval dates of drugs within this group (February 2015 and December 2014, respectively). Given that the availability of commercial antimicrobial susceptibility tests (ASTs) often lags drug approval by months to years (13), it is not surprising that CZA and C/T would have greater testing availability than more recently approved drugs. A 2021 survey of AST availability for the other four new antibiotics included in this study (FDC, IPR, MEV, and ERV) conducted among members of the American College of Clinical Pharmacist Infectious Diseases Practice and Research Network found that approximately one-third of respondents could not access AST for these agents at all, and respondents who were able to access the tests reported that it took almost 2 hours per patient to coordinate susceptibility testing (14). Timely integration of commercial AST methods for new antibiotics is an ongoing challenge and a potential barrier to appropriate clinical use of these agents (15).

Susceptibility rates for total Enterobacterales isolates were high in this study, ranging from 98.7% for CZA to 92.5% for C/T, and generally comparable across the antibiotics tested. Comparisons among agents should be considered with caution as not all antibiotics were tested in all isolates, and cascade testing (additional susceptibility testing in isolates resistant to more commonly used drugs) may have played a factor. With this caveat in mind, differences in susceptibility rates were observed in isolates with specific resistance profiles: CZA retained high levels of susceptibility against MDR, Carb-NS, and CARB-NS + MDR Enterobacterales isolates, while MVB had slightly lower susceptibility rates and C/T had markedly lower rates. There were <30 resistant Enterobacterales isolates with data for FDC, ERV, or I-R, so susceptibilities to those drugs were not reported. Varying levels of susceptibility were also observed in *P. aeruginosa* resistance profiles, although the differences were not as large. For resistant *P. aeruginosa* isolates, FDC had the highest susceptibility rates followed by C/T and CZA. Susceptibility rates generally remained consistent across different culture sources, but Enterobacterales respiratory isolates had a lower susceptibility to C/T. A recent study found that Enterobacterales isolates from pneumonia cultures had a higher proportion of resistant phenotypes (carbapenem resistance, MDR, and extensively drug-resistant) than other types of infection, which may have contributed to the lower susceptibility of C/T compared with CZA in our study (16). Carb-NS *P. aeruginosa* blood isolates had a lower susceptibility to CZA versus C/T. However, the CZA analysis included only 32 isolates, only slightly over the threshold of 30 isolates used in these analyses, which may have affected the accuracy of this evaluation.

Modest decreases in carbapenem-non-susceptible Enterobacterales susceptibility rates were observed between 2020 and 2022 for both CZA and C/T. In a surveillance study of 74 US hospitals, Sader et al. found that the proportion of carbapenem-resistant Enterobacterales isolates producing metallo-beta-lactamases increased markedly from 2019 to 2021 (16). It is possible the decreased susceptibility rates observed in our study were related to this trend, as both CZA and C/T have limited activity against metallo-beta-lactamases (8). In contrast to the trend in Enterobacterales, we observed increases in CZA and C/T susceptibility rates in carbapenem-non-susceptible *P. aeruginosa* during this time period. It is possible that these temporal changes were influenced by the COVID-19 pandemic, which was associated with increases in infections caused by AMR Enterobacterales and multidrug-resistant *P. aeruginosa* in US hospitals (17, 18). Additional studies will be required to monitor trends in susceptibility over time.

The testing and susceptibility patterns observed in this study are generally consistent with the most recent Infectious Diseases Society of America recommendations for the management of carbapenem-resistant Enterobacterales and *P. aeruginosa* with difficult-to-treat resistance (19). For most carbapenem-resistant Enterobacterales infections, CZA, MVB, and I-R are considered preferred treatment options; FDC and ERV are options under some circumstances. For difficult-to-treat *P. aeruginosa*, C/T, CZA, and I-R are generally the preferred options, with FDC as an alternative treatment option (19).

Our real-world data support the high *in vitro* susceptibility rates reported in surveillance studies for these newer antibiotics (9 – 11). Although our study did not evaluate clinical use or outcomes, studies of real-world use of these drugs have found encouraging clinical success rates, particularly given the severity of illness in many of the treated patients (20 – 25).

Limitations to this study include potential differences in breakpoints among facilities. For instance, the FDA susceptibility breakpoint for FDC in analyses of *P. aeruginosa* isolates is ≤1 µg/mL, whereas the Clinical and Laboratory Standards Institute (CLSI) has a breakpoint of ≤4 µg/mL for this drug (26). In addition, facilities tend to underreport Carb-NS Enterobacterales and *P. aeruginosa* compared with current CLSI breakpoints (27). Such differences might have influenced the proportion of susceptible isolates observed in this study and the proportion of isolates included in Carb-NS and MDR analyses. Different facilities used different antimicrobial susceptibility testing methods; although minimal inhibitory concentration (MIC) testing was the most common (>95% of isolates), disk diffusion was used for a small number of isolates and all FDC results. Susceptibility determinations in our study were based on facility interpretations rather than on exact values for MICs. Not all antibiotics were tested against all isolates, as is common in real-world use, so comparisons of susceptibility should be considered with care. Surveillance samples were excluded from these analyses, but it is possible that some isolates were not associated with a clinically significant infection. Because this study was based on data available in the laboratory information system, mechanisms of resistance were not explored, and beta-lactamase and carbapenemase production was not assessed.

Our data indicate that newer antibiotics are an important option for the management of resistant Enterobacterales and *P. aeruginosa*. Although the frequency of testing Gram-negative pathogens for CZA and C/T susceptibility appears to be increasing, the other antibiotics in this study were tested against fewer than 2% of isolates, which may contribute to the under-utilization of these drugs. The expanded use of rapid diagnostic techniques may be necessary to optimize the use of these antibiotics.

## MATERIALS AND METHODS

### Study design

We conducted a multicenter, retrospective cohort analysis of hospitalized patients with facility-reported antibiotic susceptibility results from US facilities in the BD Insights Research Database (Becton, Dickinson and Company, Franklin Lakes, NJ), which includes both small and large medical care facilities in rural and urban areas throughout the United States. This electronic surveillance system and clinical research database have been previously described and encompass pharmacy, laboratory, administrative data, patient demographics, and admission, discharge, and transfer data feeds (27 – 29). The retrospective, de-identified data set was approved and informed consent requirements were waived by the New England Institutional Review Board (Wellesley, MA, USA; IRB No. 120180023).

Eligible patients were adults (≥18 years old) hospitalized at US acute care facilities in the BD Insights Research Database between January 2018 and December 2022 with a positive first non-duplicate culture for Enterobacterales or *P. aeruginosa* from respiratory, blood, urine, intra-abdominal, skin/wound, and other sources and facility-reported antibiotic susceptibility results for at least one of the six evaluated antibiotics (see below). Surveillance cultures were excluded from analysis by a previously described algorithm (30). Analyses included both community- and hospital-onset cultures.

### Antibiotic susceptibility assessments

The antibiotic susceptibilities analyzed in this study involved newer antibiotics known to have activity against Gram-negative pathogens, specifically FDC, CZA, C/T, ERV, I-R, and MVB. Susceptibility results and interpretations (susceptible, intermediate, or resistant) were provided by the facilities based on each facility’s choice of antimicrobial susceptibility testing methods (disk diffusion or dilution) and susceptibility breakpoints; a central testing laboratory was not involved. All FDC antimicrobial susceptibility assessments were based on disk diffusion results. Carb-NS was defined as intermediate or resistant susceptibility to meropenem, doripenem, imipenem (excluding *Morganella morganii, Proteus mirabilis, and Providencia stuartii*), or ertapenem (excluding *P. aeruginosa*). MDR was defined as a categorization of intermediate (I) susceptibility or resistance (R) to ≥1 drugs in at least three of the following classes: extended-spectrum cephalosporins, carbapenems, piperacillin–tazobactam, fluoroquinolones, and aminoglycosides. As recommended in AST guidelines, susceptibility analyses were limited to those with at least 30 isolates to improve statistical validity of the estimates (12).
